# Transcription shifts in gut bacteria shared between mothers and their infants

**DOI:** 10.1038/s41598-022-04848-1

**Published:** 2022-01-24

**Authors:** Tommi Vatanen, O. Sakwinska, B. Wilson, S. Combremont, W. S. Cutfield, S. Y. Chan, K. M. Godfrey, NiPPeR Study Group, O. Sakwinska, W. S. Cutfield, S. Y. Chan, K. M. Godfrey, J. M. O’Sullivan, Sheila J. Barton, Mary Cavanagh, Yap Seng Chong, Paula Costello, Vanessa Cox, Sarah El-Heis, Mrunalini Jagtap, Karen Lillycrop, Heidi Nield, Gernalia Satianegara, Irma Silva-Zolezzi, Shu E. Soh, Gladys Woon, Tim Kenealy, Mark Vickers, Jonathan Swann, Justin M. O’Sullivan

**Affiliations:** 1 Liggins Institute, The University of Auckland, Private Bag 102904, Auckland, New Zealand; 2 The Broad Institute of MIT and Harvard, Cambridge, MA USA; 3 Nestlé Institute of Health Sciences, Nestlé Research, Société des Produits Nestlé S.A., 1000 Lausanne, Switzerland; 4 A Better Start – National Science Challenge, Auckland, New Zealand; 5 Department of Obstetrics and Gynaecology, Yong Loo Lin School of Medicine, National University of Singapore, Singapore, Singapore; 6 Singapore Institute for Clinical Sciences, Agency for Science, Technology and Research, Singapore, Singapore; 7 NIHR Southampton Biomedical Research Centre, University Hospital Southampton NHS Foundation Trust and University of Southampton, Southampton, UK; 8 MRC Lifecourse Epidemiology Centre, University of Southampton, Southampton, UK; 9 The Maurice Wilkins Centre, The University of Auckland, Auckland, New Zealand; 10 Brain Research New Zealand, The University of Auckland, Auckland, New Zealand; 11 National University Hospital, Singapore, Singapore; 12 Faculty of Medicine, University of Southampton, Southampton, UK; 13 Faculty of Biomedical Sciences, University of Southampton, Southampton, UK; 14 Nestlé Research, Société des Produits Nestlé S.A., Singapore, Singapore

**Keywords:** Metagenomics, Microbial ecology, Microbiome, Microbial communities

## Abstract

The infant gut microbiome contains a portion of bacteria that originate from the maternal gut. In the infant gut these bacteria encounter a new metabolic environment that differs from the adult gut, consequently requiring adjustments in their activities. We used pilot community RNA sequencing data (metatranscriptomes) from ten mother-infant dyads participating in the NiPPeR Study to characterize bacterial gene expression shifts following mother-to-infant transmission. Maternally-derived bacterial strains exhibited large scale gene expression shifts following the transmission to the infant gut, with 12,564 activated and 14,844 deactivated gene families. The implicated genes were most numerous and the magnitude shifts greatest in *Bacteroides* spp. This pilot study demonstrates environment-dependent, strain-specific shifts in gut bacteria function and underscores the importance of metatranscriptomic analysis in microbiome studies.

## Introduction

Large-scale microbial colonization of the infant gut begins at birth^1–3^ and these early microbial residents contribute to host development and health. For example, many of these initial inhabitants interacting with immune cells^4,5^, metabolise complex sugars (human milk oligosaccharides, HMOs) in breast milk^6–8^ and reduce the intestinal pH to restrict the growth of opportunistic pathogens^9^. The absence of bacteria with such core functions may therefore have lasting health consequences for the human host.

A portion of the pioneering infant gut bacteria are vertically transmitted from the maternal gut at birth^10,11^. The metabolic environment of the infant gut is markedly different to that of the adult gut, due to the sole reliance on breast milk and/or infant formula. Maternally derived bacteria are therefore likely to adjust their gene expression to survive in this new environment. Indeed, an initial investigation highlighted high-level functional shifts, including decreased bacterial fermentation, following mother-to-infant transmission^12^. Previous studies have not, however, investigated changes in expression of individual genes nor strain-specific transcription adjustments in maternally-derived bacterial strains.

Here, we used gut metagenomes and metatranscriptomes from 10 mother-infant dyads from the United Kingdom and New Zealand participating in the NiPPeR Study^13^ to investigate variations in the gene expression of gut bacterial strains shared between mother and infant. We specifically asked to what degree did the shared strains differ in terms of their gene expression in the infant vs. maternal gut and whether any bacterial species were more proficient in adjusting to their new environment in the infant gut.

## Results

### Gut bacteria differed taxonomically in NiPPeR mothers and their infants

The NiPPeR double-blinded, randomized controlled trial recruited 1,729 women prior to conception and followed 585 mother-infant dyads from delivery to examine the impact of a nutritional supplement [containing additional micronutrients, myo-inositol and probiotics—*Lactobacillus rhamnosus NCC 4007* (CGMCC 1.3724; LPR) and *Bifidobacterium animalis* sp. *lactis NCC 2818* (CNCM I-3446; Bl818)] taken before and during pregnancy on the health of mothers and their infants^13^. In this pilot study, we analysed 20 stool samples, collected six months after birth, from 10 mother-infant dyads within the NiPPeR cohort (five from Auckland, New Zealand; five from Southampton, United Kingdom) with appropriate positive and negative control samples to characterize vertical mother-to-infant microbial transmission and subsequent shifts in bacterial transcription (Table S1).

**Figure 1:**
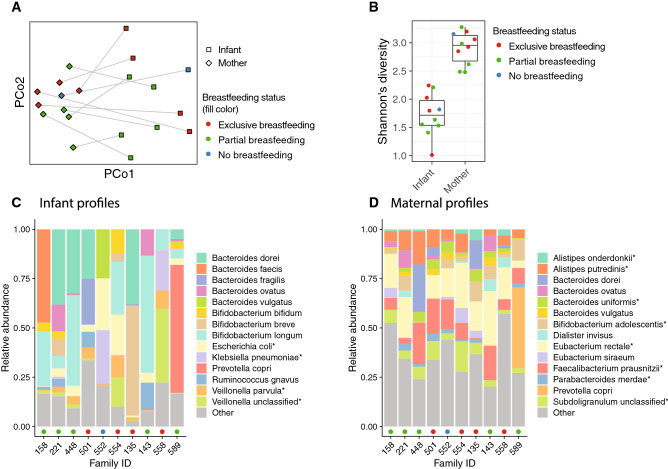
Distinctive microbial profiles separated infants from their mothers. (**A**) Principal Coordinate Analysis ordination of bacterial taxonomic profiles. Samples from the same family are connected by line. (**B**) Shannon’s diversity of microbial species profiles. The box of the boxplot shows the interquartile range, whiskers show the range of data and horizontal line in the box shows the median. (**C**–**D**) Relative abundances of the 14 most abundant species (on average) in the infant (**C**) and maternal (**D**) stool samples. Coloured points on the x-axis indicate breastfeeding status as on panel B.

We first characterised the fecal microbiomes using metagenomic DNA sequencing and investigated the microbial populations taxonomically at the species level. As expected, mothers and infants harbored distinctive microbial communities separated on Principal Coordinate Analysis ordination (Fig. 1A, PERMANOVA test R^2^ = 0.314, *p* < 0.001), mirrored by a contrasting difference in microbial diversity (Fig. 1B, Student’s t-test *p* = $1.1 \times 10^{ - 5}$). The most abundant species in infants included several species from the genera *Bifidobacterium* and *Bacteroides* as well as opportunistic pathogens such as *Escherichia coli* and *Klebsiella pneumoniae* (Fig. 1C), consistent with other infant cohorts. Mothers harbored more diverse microbial communities with many species characteristic of more complex diets such as *Faecalibacterium prausnitzii* and *Prevotella copri* (Fig. 1D)^14,15^. Analysis of breastfeeding status was underpowered and did not identify any apparent, consistent effects on the taxonomic profiles (Fig. 1A–C).

### Microbial metabolic functions differed between mothers and infants

**Figure 2:**
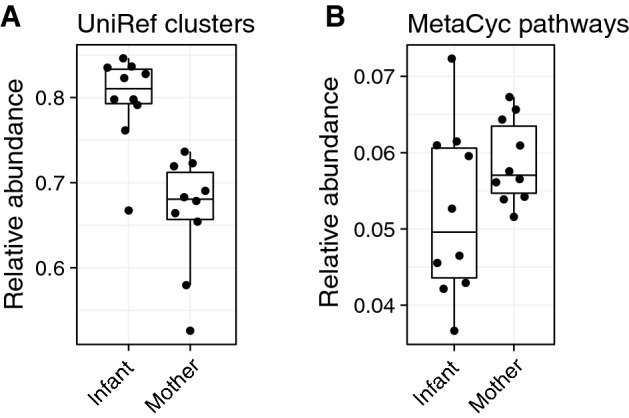
Mapping metagenomic DNA to protein databases and MetaCyc pathways. (**A**) Larger proportion of DNA reads from the infant metagenomes were mapped against UniRef protein clusters compared to adult metagenomes (Wilcoxon test, *p* = 0.0003). (**B**) Proportion of mapped DNA reads in (**A**) that also contributed to quantifications of MetaCyc pathways. The difference between mothers and infants was not statistically significant (Wilcoxon test, *p* = 0.16).

We next asked how these apparent taxonomic differences were reflected in bacterial gene abundances related to metabolic pathways in the MetaCyc database. To this end, we first compared the overall reference database coverage between adult and infant metagenomes and found that a higher proportion of infant metagenomes mapped to our reference protein database (UniRef protein clusters) compared to the adult metagenomes (*p* = 0.0003, Fig. 2A). Paradoxically, on average, a smaller proportion of the mapped reads in infants tended to have confident functional annotations in the MetaCyc pathway database, although this difference was not statistically significant (*p* = 0.16, Fig. 2B). This indicates that the bacterial genome databases cover the infant gut bacteria better than a more diverse set of bacteria harbored by adult humans but, counterintuitively, genes on the infant gut reference genomes were more poorly annotated with metabolic functions compared to adult gut genomes. Metatranscriptomic data provides additional information to prioritize such unannotated genes for in vitro functional characterization.

We then tested 354 MetaCyc pathways observed in at least two samples for differences in DNA abundance between mothers and infants and found that 152 pathways (42.9% of tested pathways) were differentially abundant between mothers and infants (linear mixed effects model, q-value < 0.05, Table S2). Of these, 104 were more abundant in infants and 48 were more abundant in mothers. We went on to confirm whether these differences persisted in the metatranscriptomic data (RNA sequencing) and quantified MetaCyc pathways by transcript (RNA) abundance. Of the slightly higher number of metabolic pathways detected by their transcripts in at least two samples (385 MetaCyc pathways), only 40 (10.4%) were differentially abundant between mothers and infants (q-value < 0.05, Table S2). For example, pyrimidine deoxyribonucleotide biosynthesis (MetaCyc identifier PWY-6545) and purine ribonucleosides degradation (PWY0-1296) were more abundant in mothers by DNA abundance (q = 9.8 × 10^–4^ and q = 7.4 × 10^–4^, respectively; Table S2) but equally abundant in mothers and infants by RNA abundance (q = 0.63 and q = 0.87, respectively). The number of differentially abundant pathways being markedly lower in the metatranscriptomic (n = 40 pathways) data compared to the metagenomic data (n = 152 pathways) could indicate that the bacterial communities were compensating for variation in DNA gene abundance by collectively adjusting gene expression to maintain diverse and stable community activity profiles.

### Gut bacterial strains shared between mothers and infants

**Figure 3:**
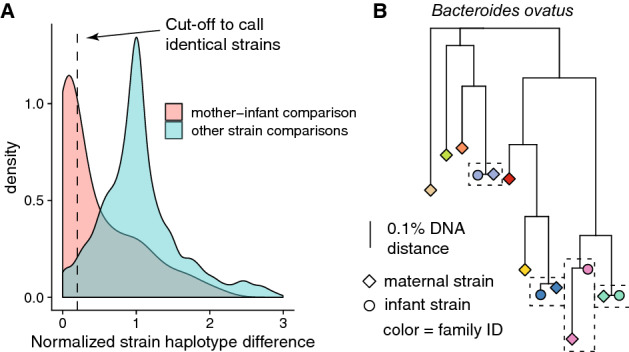
Bacterial strains shared between a mother and her infant. (**A**) Density plot of within-species strain comparisons between all detected strains per species. X-axis shows median normalized SNP-haplotype DNA distance (Jukes-Cantor model) divided by the median DNA distance per species (0 = equal strain haplotypes, 1 = median SNP haplotype dissimilarity). Red area shows comparisons within mother-infant pairs (given that a strain was detected from both infant and mother), teal area shows all other strain comparisons representing strain comparisons between random gut microbiomes. (**B**) Phylogenetic tree of *Bacteroides ovatus* strains detected in this cohort. Colors represent different mother-infant pairs (families) and scale shows the branch length corresponding to 0.1% DNA dissimilarity. Four family-specific strains are highlighted with dashed boxes.

Maternal gut strains vertically transmitted at birth contribute to the seeding of infant gut microbial communities^10,11^. We sought to identify such vertically transmitted, or otherwise shared strains between mother and infant using a single nucleotide polymorphism (SNP)-based haplotyping approach. This approach assigned a SNP haplotype profile for the dominant strain of each species in all samples. We then compared these SNP haplotypes within species and median-normalized these nucleotide identities to account for differences in population diversity between species. Family-specific mother-infant strain pairs were, on average, more similar compared to random strain pairs (Fig. 3A). We then assigned a conservative threshold of 0.2 median-normalized DNA distance to identify shared strains (Fig. 3A). Using this threshold, we found 51 bacterial strains that were shared between mothers and their infants, representing 30 different bacterial species and seven bacterial genera (Table 1). Most shared strains (36/51, 70.6%) were species in the genus *Bacteroides* and six (11.8%) were bifidobacteria. Family-specificity of the shared strains was evident, as exemplified on the strain phylogeny of *Bacteroides ovatus* with the highest number of strains (n = 4) shared between a mother and her infant (Fig. 3B). Given the maternal gut microbiome is a known source of strains colonizing the infant gut, these shared strains most likely represent vertical mother-to-infant transmission events but could also be independently acquired from other sources by both the mother and the infant, or even transmitted from the infant to the mother.

**Table 1:** Number of mother-infant pairs that shared a strain per bacterial species.

Bacterial species	Number of transmission events	Events by breastfeeding status at 6 months		
		Exclusive	Partial	None
*Bacteroides ovatus*	4	0	4	0
*Bacteroides dorei*	3	1	2	0
*Bacteroides sp 3 1 33FAA*	3	1	1	1
*Bacteroides sp 3 1 40A*	3	1	1	1
*Bacteroides sp 4 3 47FAA*	3	1	1	1
*Bacteroides sp D1*	3	0	3	0
*Bacteroides caccae*	2	0	2	0
*Bacteroides sp 2 1 16*	2	2	0	0
*Bacteroides sp 2 1 7*	2	1	1	0
*Bacteroides sp 9 1 42FAA*	2	1	1	0
*Bifidobacterium adolescentis*	2	0	2	0
*Bifidobacterium longum*	2	0	2	0
*Parabacteroides distasonis*	2	1	1	0
*Parabacteroides sp 20 3*	2	1	1	0
*Bacteroides cellulosilyticus*	1	1	0	0
*Bacteroides fragilis*	1	1	0	0
*Bacteroides sp 1 1 30*	1	0	1	0
*Bacteroides sp 2 1 22*	1	0	1	0
*Bacteroides sp 2 2 4*	1	0	1	0
*Bacteroides sp 4 1 36*	1	0	1	0
*Bacteroides thetaiotaomicron*	1	1	0	0
*Bacteroides uniformis*	1	0	1	0
*Bacteroides vulgatus*	1	0	0	1
*Bifidobacterium bifidum*	1	0	1	0
*Bifidobacterium sp 12 1 47BFAA*	1	0	1	0
*Clostridium sp SS2 1*	1	1	0	0
*Collinsella aerofaciens*	1	1	0	0
*Lachnospiraceae bacterium 5 1 63FAA*	1	1	0	0
*Parabacteroides merdae*	1	0	1	0
*Roseburia inulinivorans*	1	1	0	0

### Strains shared within a family adjust by changing gene expression

**Figure 4:**
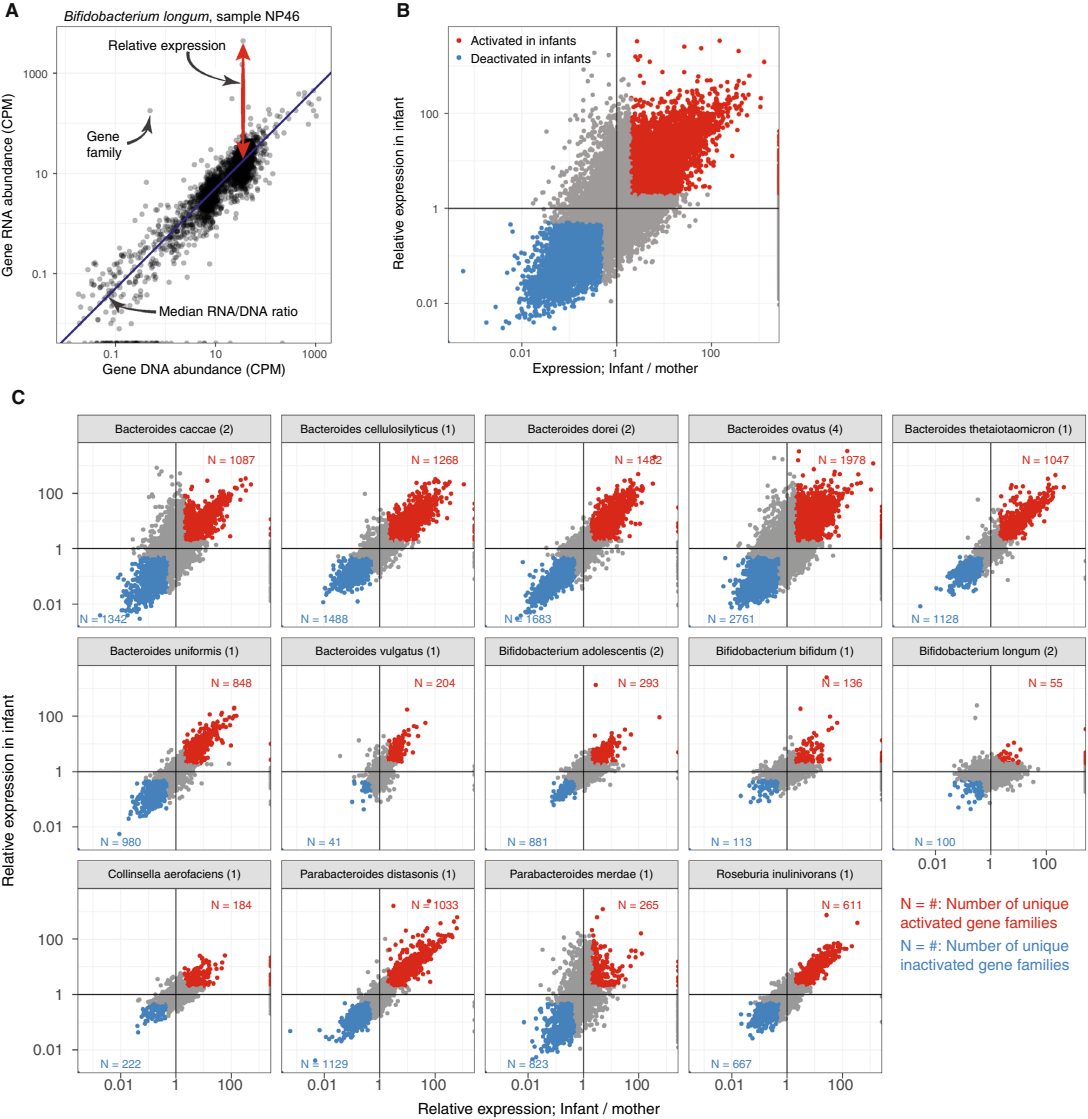
Gene expression changes in the strain shared within a family. (**A**) DNA and RNA abundances in copies-per-million (CPM) in *Bifidobacterium longum* in an infant sample (NP46). Gene family relative expression is calculated by normalizing the RNA/DNA ratio with the species and sample specific median RNA/DNA ratio. (**B**) A scatterplot of gene family relative expression change in infants vs. mothers (x-axis) and infant relative expression (y-axis), The gene families that were activated or inactivated after mother-to-infant transmission are highlighted in red and blue, respectively; activation denotes to infant relative expression > 2 (y-axis) and Infant/mother relative expression > 2 (x-axis); deactivation denotes to infant relative expression < ½ and infant/mother relative expression < ½. (**C**) Panel (**B**) stratified by species. Numbers in parentheses following the species name shows the number of families sharing a strain in the given species and panel-specific gene family frequencies (N = …) denote the number of unique gene families that were activated or deactivated in red and blue, respectively (if the same gene family was activated in multiple infants it was counted only once).

Gut bacteria respond to environmental changes through, for example, environmental and quorum sensing mechanisms. Since the environments in maternal and infant gut differ, we next investigated transcriptome (RNA) changes in the strains that were shared within a family. We detected a transcriptome signal in both maternal and infant samples for 21 of 51 (40%) shared strains with the total of 68,850 gene families, ranging between 959—6,393 unique gene families per strain per sample. We then compared gene family-specific RNA abundances, measured in copies-per-million, to corresponding DNA abundances (Fig. 4A). Since both measurements are relative (not absolute) quantifications, the average amounts of RNA and DNA detected from any given species may vary significantly. For example, if one species in a community is transcriptionally highly active (i.e. there are more RNA reads from that species compared to DNA reads), the amounts of RNA reads from all other species/strains will appear depleted compared to DNA. Within the shared strains, median RNA/DNA ratio per species per sample varied between 0.38 and 1.08. We therefore median-normalized the RNA/DNA ratio of each species sample combination and refer to this normalized RNA/DNA ratio as *relative expression*. Figure 4A illustrates the normalization for *B. longum* in one infant sample (NP46).

Since our ultimate goal was to investigate transcriptome changes between maternal and infant gut bacteria, we also measured the change in the relative expression between mothers and infants within the family (Fig. 4B, x-axis). This corresponded to asking whether a gene family on a shared strain was activated in the infant gut in comparison to how it was expressed in the maternal gut. As the analyses lacked sufficient statistical power to assess strain-specific gene expression changes, we applied thresholds in expression quantifications (relative expression in infants, ratio of relative expressions in infants and mother) to identify tentative gene activation and inactivation events. We identified 12,574 gene family activation events (18.3% of all gene families harbored on the shared strains) where the gene families were overexpressed in infants (relative expression, RNA/DNA > 2) and had at least twofold higher relative expression in infants compared to their mothers (Fig. 4B, red points; Table S3). Slightly more gene families, n = 14,844 (21.6%), were deactivated in infants (relative expression < 0.5) and had at least twofold higher relative expression in mothers (Fig. 3B, blue points; Table S3). Stratifying these findings by bacterial species revealed marked differences in the frequency of (de)activated gene families and the changes in relative expression (Fig. 4C). Many species in the genus *Bacteroides* activated and deactivated more than one thousand genes following tentative mother-to-infant transmission, with many gene families showing an over 100-fold increase in relative expression between the infant and the mother. While we also detected (de)activated gene families in bifidobacteria, the frequency and magnitude of the changes in bifidobacteria were less pronounced (Fig. 4C). For example, *Bifidobacterium longum*, known for its versatile breast milk metabolism, only activated 55 unique gene families in two tentative mother-to-infant transmission events. Stratifying these observations by breastfeeding status was underpowered and did not show any consistent trends (Table S3). Overall, of 68,850 gene families harbored by the shared bacterial strains, 37,156 (54.0%) did not have any homology-based function annotation (i.e. were unannotated), whereas 5,764 of 12,564 (45.9%, strain-by-strain paired Wilcoxon test, *p* = 9.5 × 10^–7^) activated genes and 9,935 of 14,844 (66.9%, *p* = 9.5 × 10^–6^) deactivated genes were unannotated.

Finally, we asked whether the gene families activated in infants were consistent between mother-infant pairs using data from three *Bacteroides* and two *Bifidobacterium* species which had shared strains in more than one mother-infant pair. This “replication rate” was highest in *Bacteroides dorei* and *Bacteroides ovatus*, where 46.2% and 45.7% of the infant-activated gene families were consistent in two or more infants, respectively. In *Bifidobacterium adolescentis* and *Bifidobacterium longum* only 3.4% and 1.8% of activated gene families were consistent between multiple infants.

## Discussion

We demonstrated differences in functional activity and strain-specific gene expression in the gut microbiomes of ten mother-infant dyads. Several strains from the genera *Bacteroides* and *Bifidobacterium* that were shared between mother-infant pairs showed marked adjustments to the infant gut environment by thousands of gene (de)activation events. The magnitudes of these shifts were largest in *Bacteroides* spp. which also contribute to the metabolism of dietary carbohydrates, including oligosaccharides in breast milk^16–18^. Larger, longitudinal studies are needed to assess reproducibility and temporal stability of such bacterial adjustments in the infant gut.

The relative abundance of metagenomic gene families that could be mapped to metabolic pathways in the MetaCyc database was lower in infants compared to their mothers. Mirroring this, roughly half of the genes tentatively contributing to bacterial adjustments were unannotated providing another priority research question; what are these genes doing? Metatranscriptomic data will be useful for prioritizing bacterial genes and their protein products for in vitro functional characterization. Priority could be given, for example, to genes that are highly expressed in infants, are widely prevalent (present in most infants) or infant-specific, are associated with important infant phenotypes or are harbored by bacteria of interest (e.g. bifidobacteria); or any combination of such criteria. New computational annotation methodologies and gut metabolite profiles could also provide insights into the functions and metabolic targets/outputs of these genes in a less targeted manner.

This pilot study included data from ten mother-infant pairs, with one stool sample per participant, and is therefore limited in statistical power. Additionally, our study design did not allow us to evaluate the null distribution of bacterial gene expression changes in an individual (a mother or an infant) over time. In addition to including more participants, future studies should preferably incorporate multiple stool samples from both mothers and infants to assess and quantify day-to-day fluctuations in bacterial gene expression. This would enable identification of gene expression shifts in maternally-derived strains that are statistically different from day-to-day variations. Optimally, longitudinal sampling in infants should also cover the transition from exclusive breastfeeding to introduction of solid foods and weaning to investigate how this common early feeding pattern affects microbial transcription patterns. The gene expression shifts in *Bifidobacterium* spp. and *Bacteroides* spp., for example, could be more pronounced during exclusive breastfeeding and then stabilize towards adult profiles after weaning. Finally, larger studies could more robustly assess species-specific replication rates of gene expression changes in specific gene families and whether the same genes are consistently up- or down-regulated across individuals.

We focused on bacterial species tentatively transmitted from the maternal to infant gut yet a large proportion of infant gut bacteria are obtained from other sources^10^. Future studies may therefore fruitfully explore gene expression and metabolic adjustments of bacteria obtained from other, ecologically more distant environments, since such adjustments may require even more radical activity alterations. Researchers should also investigate whether and to what degree bacterial transcription and its adjustments play a role in their engraftment capacity and early gut microbiome assembly.

## Conclusions

We observed large scale gene expression shifts in gut bacterial strains shared between mothers and their infants. These gene expression shifts were most prominent in *Bacteroides* spp. potentially reflecting bacterial adjustments to the new environment. Taken together, metatranscriptomic data provides an important ‘omics data layer adding information on environment-dependent community metabolic activities and adjustments to microbiome investigations.

## Methods

### Trial registration and ethics approval

NiPPeR is an academic-led study by the EpiGen Global Research Consortium (ClinicalTrials.gov NCT02509988, Universal Trial Number U1111-1171-8056; 16/7/2015, https://www.clinicaltrials.gov/ct2/show/NCT02509988). After independent, full, external peer review the study protocol and subsequent amendments have been approved by the Research Ethics Committees at each of the three study sites (Southampton: Health Research Authority NRES Committee South Central Research Ethics Committee (REC), reference 15/SC/0142), the Health and Disability Ethics Committee (HDEC) (New Zealand) reference 15/NTA/21 and the National Healthcare Group Domain Specific Review Board (NHG DSRB) (Singapore) reference 2015/00205). This study is being conducted in compliance with the NiPPeR study protocol, Good Clinical Practice and the applicable regulatory requirements. All data analysed in this study was stored and analysed in accordance with the UK Data Protection Act, and applicable regulations and guidance of each country and institution.

### Sample collection and preservation

Stools were collected from mothers and infants in Southampton and New Zealand according to a standardised S.O.P to ensure uniformity (6SOP7, Stool [collection and processing], effective date 13/08/2016, Revision V1.0). Briefly, on the sixth month post-delivery clinical visit, mothers were given a bedpan liner (Vernacare) and asked to (1) pass urine into the toilet prior to placing the tray on the toilet seat; (2) pass the stools; (3) place a small scoop into the collection tube (Sarstedt Faeces container with screw cap, polypropylene, sterile, Size: 101 mm [L] × 16.5 mm [Dia.]) containing RNAlater Stabilisation Solution (5 ml). Participants were instructed to screw down the cap tightly and shake vigorously at least 10 times to ensure that the sample has been well-mixed into the solution. Infant samples were taken directly from the nappy and placed in collection tubes containing RNAlater, as per the maternal samples. The time and date of the collection were immediately recorded on the tube and specimen containers placed on ice and taken to the laboratory. If collected at home, samples were held at −20 °C until delivery to the clinic.

Fecal samples in the RNAlater tubes were processed [placed in a Falcon tube, centrifuged (4600 rpm, 4 °C, 5 min)] within a maximum of 60 min from receipt of samples from subject (start and end times were recorded). RNAlater was discarded and 0.5 g of sample was transferred into each of 6 × 3 ml micronics tubes. Sample weights of each aliquot were recorded and samples stored (− 80 °C) until analysis.

### DNA extraction controls

Negative and positive DNA extraction controls were extracted and included in the analysis. The negative control was 200 µl RNase free water. Two microbial community standards were used as positive controls; (1) 20 Strain Even Mix Whole Cell Material (ATCC MSA-2002, Virginia, USA) embedded in a stool matrix (#NATROTA-GP, ZeptoMetrix Corporation, New York, USA), and (2) ZymoBIOMICS Microbial Community Standard (#D6300, Zymo Research, California, USA).

### Nucleic acid extraction and library preparation

DNA and RNA extractions were performed at the Liggins Institute (University of Auckland, New Zealand) using a modified protocol of the AllPrep DNA/RNA Mini Kit (#80204, Qiagen, Germany) as described previously^19^. Briefly, stool aliquots were thawed and incubated in 100 µl of lysis buffer (30 mM Tris–HCl, 1 mM EDTA, 15 mg/ml lysozyme) and 10 µl proteinase K for 10 min at room temperature with regular agitation. Samples were then mixed with 1.2 ml RLT plus buffer (Qiagen, Germany), 12 µl beta-mercaptoethanol, and 1 ml of acid washed glass beads (≤ 106 µm, − 140 U.S. sieve; #G4649-100G, Sigma Aldrich, USA) and shaken vigorously at 30 Hz for 10 min on a TissueLyser II (Qiagen, Germany). The homogenate was then passed through a QIAshredder spin column (#79654, Qiagen, Germany), before continuing with the standard AllPrep DNA/RNA Mini kit protocol (#80204, Qiagen, Germany). DNA was eluted in 75 µl of EB buffer and RNA eluted in 50 µl RNase free water. DNA was quantified by Qubit dsDNA High Sensitivity Assay Kit (#Q32851, Invitrogen, California, USA). RNA was quantified by Qubit RNA High Sensitivity Assay Kit (#Q32852, Invitrogen, California, USA) and quality assessed using Agilent RNA 6000 Nano Kit and Agilent 2100 Bioanalyzer instrument (Agilent technologies, California, USA). Sample data (including RNA integrity Number, RIN) is available in Table S1.

### DNA shotgun sequencing

DNA sequencing was performed following a previously described procedure^20^. Genomic DNA was fragmented (random shearing) into ~ 350 bp fragements. Sequencing libraries were prepared from the fragmented DNA using the NEBNext Ultra Library Prep Kit for Illumina (New England Biolabs). Fragment size distribution within the prepared sequencing libraries was evaluated (Qubit 2.0 fluorometer quantitation and Agilent 2100 Bioanalyzer). Prior to sequencing, DNA concentrations of the sequencing libraries were measured by quantitative real-time PCR (qPCR). Sequencing libraries were sequenced on an Illumina platform (2 × 150 bp paired-end sequencing).

### RNA shotgun sequencing

The Ribo-Zero kit was used to deplete samples of rRNA before random fragmentation of the mRNA. cDNA synthesis was performed according to^21^. cDNA was synthesized from mRNA using random hexamers primer, after which a custom second-strand synthesis buffer, dNTPs, RNase H and DNA polymerase I were added to initiate the second-strand synthesis. After terminal repair, A ligation, and sequencing adaptor ligation, the double-stranded cDNA library was completed through size selection and PCR enrichment. The cDNA library was sequenced using paired-end 150 bp sequencing.

### Bioinformatics

Both DNA and RNA sequencing data were subject to standard quality control, including read trimming based on base call qualities and host contamination removal. We obtained, on average, [mean (min–max)] 22.6 M (16.7–27.7 M) and 45.6 M (32.4–55.5 M) quality controlled, paired sequencing reads for DNA and RNA sequencing data, respectively. Bacterial species abundances were quantified using the metagenomes and MetaPhlAn2 v2.7.7^22^. Alpha-diversities of the taxonomic profiles were measured by Shannon’s diversity index and beta-diversities were measured by Bray–Curtis dissimilarity. Principal Coordinate ordination was generated using Nonmetric Multidimensional Scaling (metaMDS function in vegan R package v 2.5-6, https://cran.r-project.org/web/packages/vegan/). SNP-haplotypes of the dominant bacterial strains of a given species per sample were determined by StrainPhlAn^23^ by requiring a minimum coverage of 5 bases for SNP calling (‘–min_read_depth 5’ command line parameter for sample2markers.py Python script). The phylogenetic tree (Fig. 3B) was generated using the phangorn R package (v2.5.5, https://cran.r-project.org/web/packages/phangorn/). Species-stratified gene family abundances from both metagenomes and metatranscriptomes were quantified by HUMAnN2 v0.11.2^24^ which involves mapping sequencing reads against custom species pangenomes. Gene family abundances were further stratified to quantify MetaCyc pathways by methodology implemented in HUMAnN2. Using HUMAnN2 for both metagenomic and metatranscriptomic analyses enabled co-analysis of DNA and RNA abundances of any given gene detected by both sequencing methods.

### Statistical analysis

Homology-based functional annotations of (de)activated genes were compared to annotations of all genes harbored by the shared strains as follows. For each shared strain, percentage of annotated genes among all genes and (de)activated genes were computed. These percentages were compared using paired Wilcoxon rank sum test across all transmitted strains. For all statistical testing scenarios with multiple testing, false discovery rate corrected p-values (q-values), are reported.

### Ethics approval and consent to participate

The study protocol and subsequent amendments have been approved by the Research Ethics Committees at each of the three study sites (Southampton: Health Research Authority NRES Committee South Central Research Ethics Committee (REC), reference 15/SC/0142), the Health and Disability Ethics Committee (HDEC) (New Zealand) reference 15/NTA/21 and the National Healthcare Group Domain Specific Review Board (NHG DSRB) (Singapore) reference 2015/00205). All participating mothers gave written, informed consent at the first preconception study visit.

## Supplementary Information

Supplementary Information 1.

Supplementary Information 2.

Supplementary Information 3.

Supplementary Information 4.

## Data Availability

Sequencing data will be made publicly available in NCBI Sequence Read Archive upon publication of this manuscript under BioProject accession PRJNA707065. Analysis scripts and intermediate data products to reproduce all analyses are available at https://github.com/tvatanen/nipper-metatranscriptomics.
