# Supplementary material for: Transcription shifts in gut bacteria shared between mothers and their infants
Source: Sci Rep. 2022 Jan 24;12:1276. doi: 10.1038/s41598-022-04848-1 (PMC8786960; doi:10.1038/s41598-022-04848-1)
Supplement: Supplementary file 4 — Supplementary Information 4. [file 41598_2022_4848_MOESM4_ESM.pdf]

Supplementary material for:

Transcription shifts in gut bacteria shared between mothers and their infants: pilot data from the NiPPeR Study

Vatanen T.[1,2], Sakwinska O.[3], Wilson B.[1], Combremont S.[3], Cutfield, W.S[1,4], Chan S.Y.[5, 6], Godfrey K.M[7,8], NiPPeR Study Group[†], O'Sullivan J.M.[1,8,9,10]

[1] Liggins Institute, University of Auckland, Auckland, New Zealand
[2] The Broad Institute of MIT and Harvard, Cambridge, MA, USA
[3] Nestlé Institute of Health Sciences, Nestlé Research, Société des Produits Nestlé S.A., 1000 Lausanne, Switzerland
[4] A Better Start – National Science Challenge, Auckland, New Zealand
[5] Department of Obstetrics and Gynaecology, Yong Loo Lin School of Medicine, National University of Singapore, Singapore
[6] Singapore Institute for Clinical Sciences, Agency for Science, Technology and Research, Singapore
[7] NIHR Southampton Biomedical Research Centre, University Hospital Southampton NHS Foundation Trust and University of Southampton, Southampton, UK
[8] MRC Lifecourse Epidemiology Centre, University of Southampton, Southampton UK
[9] The Maurice Wilkins Centre, The University of Auckland, Auckland, New Zealand
[10] Brain Research New Zealand, The University of Auckland, Auckland, New Zealand
[†] A list of authors and their affiliations appears at the Supplement; consortium representative Justin O'Sullivan (justin.osullivan@auckland.ac.nz)

NiPPeR Study Group

Sakwinska O.[3], Cutfield, W.S[1,4], Chan S.Y.[5, 6], Godfrey K.M[7,8], O'Sullivan J.M.[1,8,9,10], Sheila J Barton[8], Mary Cavanagh[1], Yap Seng Chong[5,6,11], Paula Costello[8], Vanessa Cox[8], Sarah El-Heis[8,12], Mrunalini Jagtap[6], Karen Lillycrop[13], Heidi Nield[8], Gernalia Satianegara[6], Irma Silva-Zolezzi[14], Shu E Soh[6], Gladys Woon[6], Tim Kenealy[1], Mark Vickers[1], Jonathan Swann[12]

[11] National University Hospital, Singapore
[12] Faculty of Medicine, University of Southampton, Southampton, UK
[13] Faculty of Biomedical Sciences, University of Southampton, Southampton, UK
[14] Nestlé Research, Société des Produits Nestlé S.A., Singapore

Supplementary Table Legend

Table S1. Stool samples analyzed in this study including concentrations of extracted DNA and RNA, and RNA integrity Number (RIN).

Table S2. MetaCyc pathways with differences in DNA (sheet 1) or RNA (sheet 2) abundance between mothers and infants. Pathway abundance in copies-per-million were tested using linear models in MaAsLin2 with country and binary indicator for mother vs. infant as fixed effects and family ID as a random effect.

Table S3. Number of activated and deactivated genes per strain per family (sheet 1). List of genes harbored by strains shared between mother and infant that were either activated (sheet 2) or deactivated (sheet 3) in infant gut compared to the maternal gut. Activation was defined as at least 2-fold higher RNA abundance compared to DNA abundance (relative expression, RNA / DNA > 2) and at least 2-fold higher relative expression in infants compared to their mothers.

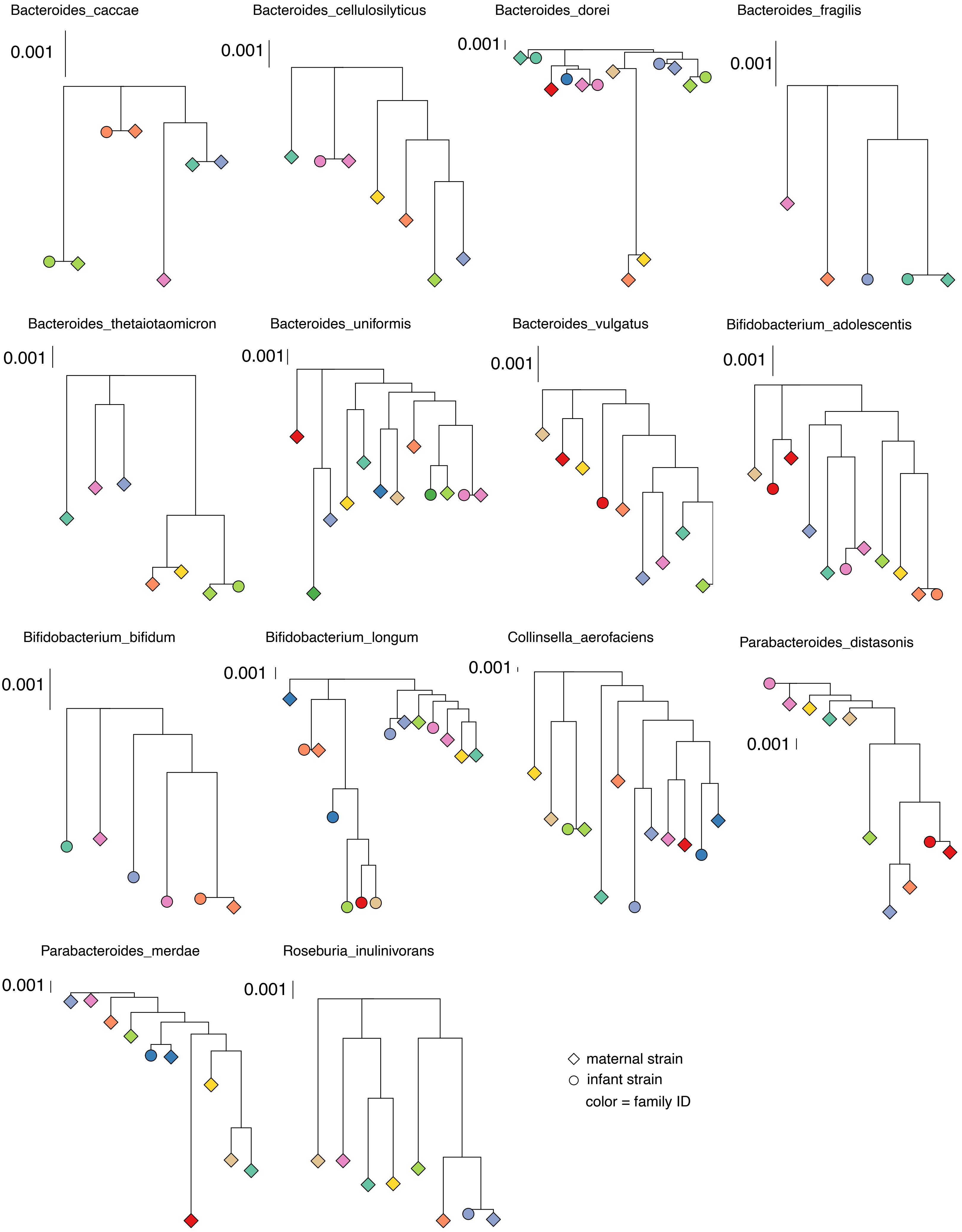
Bacteroides_caccae
0.001
Bacteroides_cellulosilyticus
0.001
Bacteroides_dorei
0.001
Bacteroides_fragilis
0.001
Bacteroides_thetaiotaomicron
0.001
Bacteroides_uniformis
0.001
Bacteroides_vulgatus
0.001
Bifidobacterium_adolescentis
0.001
Bifidobacterium_bifidum
0.001
Bifidobacterium_longum
0.001
Collinsella_aerofaciens
0.001
Parabacteroides_distasonis
0.001
Parabacteroides_merdae
0.001
Roseburia_inulinivorans
0.001
maternal strain
infant strain
color = family ID

Figure S1. Phylogenetic trees of bacteria with shared between mothers and their infants. Colors represent different mother-infant pairs (families) and scale bar shows the branch length corresponding to 0.1% DNA dissimilarity.
